# Cultural malpractice during pregnancy, childbirth, and the postnatal period and its associated factors among women who gave birth once in Dire Dawa city administration, Eastern Ethiopia, in 2021

**DOI:** 10.3389/fgwh.2023.1131626

**Published:** 2023-08-17

**Authors:** Mickiale Hailu, Aminu Mohammed, Yitagesu Sintayehu, Daniel Tadesse, Legesse Abera, Neil Abdurashid, Milkiyas Solomon, Momina Ali, Dawit Mellese, Tadesse Weldeamaniel, Teshale Mengesha, Tekelebirhan Hailemariyam, Sewmehon Amsalu, Yesuneh Dejene, Meklit Girma

**Affiliations:** ^1^ College of Medicine and Health Sciences, Dire Dawa University, Dire Dawa, Ethiopia; ^2^ College of Health Sciences, Wachemo University, Hossana, Ethiopia; ^3^ College of Health Sciences, Mekelle University, Mekelle, Ethiopia

**Keywords:** cultural malpractice, pregnancy, child birth, postnatal, women’s

## Abstract

**Background:**

Cultural practices are any experiences or beliefs that are socially shared views and behaviors practiced in a certain society at a certain time. Cultural malpractices are defined as socially shared views and traditionally accepted behaviors experienced in a certain society that harm maternal health. Worldwide, the period of pregnancy, labor, and delivery is embedded with different beliefs, customs, and rituals in different societies that contribute a lot to maternal death. They are responsible for the annual deaths of 303,000 mothers and 2.7 million newborns globally. In developing countries, it accounts for approximately 5%–15% of maternal deaths. In Ethiopia, approximately 18% of infant deaths occur due to cultural malpractice, and 52% of pregnant mothers give birth at home following cultural customs in Dire Dawa city. The objective of this study was to assess cultural malpractices during pregnancy, childbirth, and the postnatal period and its associated factors among women who gave birth once in Dire Dawa City in 2021.

**Methods:**

Community-based mixed study was conducted. A total of 624 study participants were selected through a systematic random sampling technique, and a purposive sampling method was used for qualitative data. The study was conducted in the randomly selected Kebeles of Dire Dawa City, Eastern Ethiopia, from November 1 to December 30, 2021. Data were entered into Epi Data version 4.1 and exported to SPSS version 24 for analysis. Bivariate and multivariate analyses were done, and the degree of association was measured by using the odds ratio with 95% CI and significance was declared at a *p*-value of <0.05. The qualitative data were analyzed thematically using ATLAS-ti version 7.

**Results:**

The overall prevalence of cultural malpractice during pregnancy, childbirth, and the postnatal period was 74.6% [95% CI: 70.59%, 77.49%]. Women over the age of 35 were two times more likely [AOR 2.61, 95% CI, 1.45–4.72] to commit cultural malpractice than women aged 15–24 and 25–34. Those with no antenatal care (ANC) follow-up were three times more likely to commit cultural malpractice [AOR 3.57, 95% CI, 1.72–7.40], those who were absent from health education were nearly two times more likely to commit cultural malpractice [AOR 1.83, 95%CI, 1.25–2.67], and women whose culture allows harmful traditional practices were nearly two times more likely to commit cultural malpractices than their counterparts [AOR 1.69, 95%CI, 1.29–2.54].

**Conclusion:**

In this study, nearly three-fourths of participants were involved in cultural malpractices. Therefore, strengthening community education and behavioral change messages on the importance of ANC and avoiding unhealthy care during pregnancy, childbirth, postnatal and neonatal periods, especially with pregnancy at old age (age > 35), may help to reduce cultural malpractices.

## Introduction

Cultural practices are any experiences or beliefs that are socially shared views and behaviors practiced in a certain society at a certain time. Cultural malpractices (CMPs) are defined as socially shared views and traditionally accepted behaviors experienced in a certain society that harm maternal health. Worldwide, the period of pregnancy, labor, and delivery is embedded with different beliefs, customs, and rituals in different societies that contribute a lot to maternal death. The majority of the time, those CMPs are largely carried out without the consent of the child/woman (1–4).

Globally, approximately 78 million newborns waited more than 1 h to breastfeed, and two out of five newborns were excluded from colostrum feeding. This figure translates every day to approximately 4,000 infants and young children dying due to colostrum avoidance and the introduction of pre-lacteal feeds, which are part of cultural malpractices. In general, cultural malpractices during pregnancy, delivery, and the postnatal period killed 303,000 mothers and 2.7 million newborns each year (5–7).

The World Medicine Situation report estimates that between 70% and 95% of the population in developing countries and more than 80% of the population in Africa participate in cultural malpractices during pregnancy (8, 9). The World health organization has identified sepsis as the major cause of maternal deaths in sub-Saharan Africa, which accounts for approximately 11% of maternal deaths (10).

Many mothers suffer from infection of the reproductive tract and neonatal sepsis as a result of an unsanitary environment and inadequate care during pregnancy and delivery, with infants improperly delivered by unskilled birth attendants, and/or traditional delivery practices being the leading cause of sepsis and death. The actual incidence of cultural malpractices in developing countries accounts for approximately 5%–15% of maternal deaths (9–11).

The Ethiopian government and other concerned bodies have made some efforts to combat CMPs in different parts of Ethiopia. For example, extensive health education toward the eradication of the identified cultural malpractices was delivered to society via health extension workers, and a regular health education program was provided at health facilities and community levels. In addition to that, the National Constitutional, Criminal and Family laws, Education, Health, Population, and Cultural policies of Ethiopia included articles that directly or indirectly combat CMPs. However, deep-rooted beliefs, customs, rational attitudes, lack of knowledge, and being unaware of the effects of the practices help to maintain these problems (12, 13).

In Ethiopia, more than half of pregnant mothers give birth at home and follow the cultural birth customs. Approximately 18% of infant deaths occur due to cultural malpractices during the postnatal period. According to the Ethiopian Ministry of Women, Children, and Youth Affairs report, there are various forms of CMPs against women and children, which are widely practiced in Ethiopia. But the types and prevalence of those practices vary among regions, cultural settings, and religious values. So routine assessment and investigation of CMPs are mandatory (12–14).

As seen in other parts of Ethiopia, in Dire Dawa town and around, there may be different cultural practices that are practiced during pregnancy and childbirth, but there is no data that clearly indicates the types and prevalence of cultural malpractice and their influencing factors; however, it is known that 52% of pregnant mothers give birth at home following cultural customs (15). Even though few studies have been conducted regarding cultural malpractices during pregnancy, labor, and the postpartum period, some factors like family, social, and health system factors have not been assessed yet. So, the purpose of this study was to assess the magnitude of cultural malpractices during pregnancy, childbirth, and postpartum and its associated factors among women who gave birth once in Dire Dawa city administration.

## Methods, study area, period, and design

Dire Dawa town is one of the federal city administrations in Ethiopia, which is located at a distance of 515 kilometers from Addis Ababa (the capital city) to the east. The city administration has 9 urban and 38 rural kebeles (the smallest administrative division and sometimes also called *tabia* or *tabiya*. They are at the neighborhood level and are the primary contact for most citizens living in Ethiopia). The Ethnic groups in the region include the Oromo (46%), Somali (24%), Amhara (20%), Gurage (15,543, 4.5%), and other groups (5.5%). The total fertility rate and urban unemployment rate of the city are 3.4 children per woman and 25.3%, respectively. The literacy rate for women is 50.8% and 78.6% for men.

There are 2 government hospitals, 5 private hospitals, 15 health centers, and 33 health posts. The current metro area population is 426,129, of which 49.8% of them are men and 50.2% are women. The total number of women in the reproductive age group (15–49 years) is 506,637, which accounts for 15.4% of the total population (16). A community-based quantitative cross-sectional study complemented by a qualitative study was conducted from November 1 to December 30, 2021.

## Study subjects and selection criteria

The source populations were all women who had at least one history of delivery in Dire Dawa city administration, and the study populations were systematically selected women who had experienced at least one history of delivery at the selected kebeles in Dire Dawa city administration during the study period. The study included women aged 18 to 49 who had given birth at least once in the previous 5 years and had stayed in the kebele for more than 6 months. Women who were in the immediate postpartum period and women who had chronic medical illnesses were excluded from the study.

## Sample size and sampling procedure

The sample size for the quantitative part was calculated by using the single population proportion formula with the assumption of *n* = sample size required for the study, Z*α*/2 = 1.96, which is the standardized normal distribution curve value for the 95% CI, *p* = 0.383 (9), and d = 0.04, which is the degree of freedom or margin of error.

$$n=\frac{{\left(z\right)}^{2}\times p(1-p)}{d^{2}}$$

$$=\frac{{\left(1.96\right)}^{2}\times 0.383\;(1-0.383)}{{\left(0.04\right)}^{2}}$$

n=624afteradding10%ofthenon-responserate

## The sample size for qualitative data

A total of 20 individual participants (5 Key informants (KIs) and 15 In-depth interviews (IDI) were planned to be involved in this study, but the final sample size was determined by data saturation.

## Sampling technique and data collection procedure

The Dire Dawa City administration has 9 urban and 38 rural kebeles. Using the random sampling method, five kebeles were selected. Then, based on the total number of women aged 18 to 49 who had given birth at least once in the previous 5 years in each selected kebeles, the sample size was proportionally allocated to each selected kebeles. Individual participants in each kebele were selected using a systematic random sampling technique by considering their house number as a sampling frame. The desired sampling interval was calculated using the two-month delivery reports of each health facility in each kebele (977/624 = 2). The first participant was selected by lottery. The subsequent units were selected at fixed intervals with a skip interval of k = 2.

## Data collection tools and procedures

### The quantitative part

First, the questionnaires were adopted from previous studies and were prepared in Afan Oromo, Somali, and Amharic versions. A total of five midwives with BSc were recruited for the community-based data collection, and face masks were given to the women during the process of data collection.

### The qualitative part

Qualitative data was collected through in-depth interviews and key informant interviews using semi-structured questions. A total of two midwives with BSc who have experience in qualitative data collection were recruited for data collection. A purposive sampling technique was used for the qualitative study. Women who have experienced cultural malpractices were selected for IDI. The KIs for this study were religious and community leaders. The principal investigator was moderating IDI and KI interviews and was assisted by an experienced notetaker. After the notetaker and the interviewer introduce themselves, the participants were informed about the purpose of the study and the confidentiality of the data, and probing questions were forwarded to the participants. All interviews were tape-recorded and transcribed in full text.

### Operational definitions

Cultural malpractices during pregnancy, childbirth, and the postnatal period: Involvement in one or more cultural practices during pregnancy, labor, and postpartum that the Ethiopian Ministry of Women, Children, and Youth Affairs classifies as harmful cultural malpractice (14).

### Data quality control

For quantitative data collection, before the actual data collection, data collectors obtained two-day training about the aim of the study and the content of the instrument. A pretest was done in Dire Dawa city administration (non-selected kebeles) on 5% of the sample size. For qualitative data collection, open-ended questions were used to avoid acquiescence bias, and proper training was given to the data collector regarding how to take keynotes and record using a tape recorder. For consistency and possible modification, a pre-test was done on one key note, and in-depth interviews were conducted in Dire Dawa city administration (non-selected kebeles). The recorded data was transcribed by experts. The key informant and in-depth interviews were conducted in a silent place.

### Data processing and analysis

The quantitative part was entered after the data collection into Epi Data version 4.1, and the data was exported to SPSS version 24 for analysis. First descriptive analysis was done, and then, bivariate analysis was done. In multivariate logistic regression analysis, a variable with a *p*-value <0.2 in bivariate logistic regression analysis was entered, and the point degree of association was declared at a *p*-value of 0.05. An odds ratio with a 95% confidence interval was used to measure the degree of association between those significant independent variables and cultural malpractices practiced during pregnancy, childbirth, and the postnatal period. The dependent variable was classified as a dummy variable. The model fit was checked by the Hosmer and Lemeshow Test, which is the most reliable test of model fit available in SPSS. Hence, the chi-square value for the Hosmer-Lemeshow test is 4.024 with a significance level of 0.74. This value is greater than 0.05, therefore indicating the goodness of model fit. A Multicollinearity test was also done to assess how much the variance of an estimated regression coefficient increases if the predictors are correlated. All values of the variance inflation factor were less than two (a maximum of 1.309 and a minimum value of 1.073). All values of the tolerance test were also above 0.2 (a maximum of 0.954 and a minimum value of 0.764). These values show that there was no problem with multicollinearity in this study. The results are presented in the form of narrations, tables, and graphs.

### The qualitative part

The data was analyzed thematically using computer-assisted qualitative data analysis software ATLAS-ti version 7. The transcribed data was entered into the software, and similar ideas were organized together to create codes. Then, themes were derived from the codes in the transcribed data and translated into an English version by language experts. Their inductive meaning was extracted using the verbatim of the participants. Then, the final report was developed using the narrative analysis method.

### Results

A total of 624 women who had experienced at least one history of delivery found in the selected kebeles of Dire Dawa city were included in the study, and the response rate was 100%.

### Socio-demographic characteristics of study participants

Regarding the socio-demographic characteristics of the respondents, 305 (48.9%) were in the age group of 25–34. The majority of them were from urban areas (471 or 75.5%) and married (527 or 98.7%) by residence and marital status, respectively. Out of the 624 participants, the majority of them (314 or 50.3%) had completed secondary education and above, followed by primary education (187 or 30%). Concerning the average monthly income, the majority of the participants (457 or 73.2%) had less than 1,500 Ethiopian Birr (Table 1).

**Table 1:** A socio-demographic characteristic of respondents to assess the prevalence of cultural malpractice during pregnancy, childbirth, and the postnatal period among women who gave birth once in Dire Dawa city in 2021 (*N* = 624).

Characteristics		Frequency	Percent
Age	18–24	116	18.6
	25–34	305	48.9
	35 and above	203	32.5
Residence	Urban	449	71.9
	Rural	175	28.1
Educational status	Non-formal education	123	19.7
	Primary (1–8)	187	30
	Secondary and above	314	50.3
Marital status	Married	527	84.5
	Divorced	17	2.7
	Widowed	80	12.8
Occupational status	Housewife	356	57.1
	Government employed	116	18.6
	Self-employed	152	24.4
Monthly income	Less than 1,500	457	73.2
	1,501–2,499	80	12.8
	Above 2,500	87	13.9

### Obstetrical characteristics of study participants

Concerning the obstetrical characteristics of study participants, 525 (84.1%) of the participants were not pregnant during the study period, and 519 (83.2%) of the participants had received antenatal care follow-up (ANC) during the last pregnancy. Among those who had received ANC follow-up during the last pregnancy, 388 (62.2%) of the participants had attended three to four ANC visits. Regarding the post-natal care and place of delivery of study participants during the last pregnancy, 433 (70%) of the participants had post-natal care follow-up (PNC) and 528 (84.6%) of the participants gave birth in a health facility (Table 2).

**Table 2:** Obstetrics characteristics of respondents to assess the prevalence of cultural malpractice during pregnancy, childbirth, and the postnatal period among women who gave birth once in Dire Dawa city in 2021 (*N* = 624).

Characteristics		Frequency	Percent
Pregnancy status (currently)	Yes	99	15.9
	No	525	84.1
Gravidia (*n* = 99)	Less than 2	65	10.4
	3–4	29	4.6
	Five and above	5	0.8
Parity	Less than 2	415	66.5
	3–4	154	24.7
	5 and above	55	8.8
ANC follow-up (currently or since your last pregnancy)	Yes	519	83.2
	No	105	16.8
Number of ANC follow-ups (*n* = 519)	Less than 2	57	10.1
	3–4	388	68.4
	5 and above	122	21.5
PNC follow-up (currently or since your last pregnancy)	Yes	433	69.4
	No	191	30.6
Number of PNC follow up (*n* = 433)	Less than 2	36	8.3
	3–4	92	21.2
	5 and above	306	70.5
Birth interval	Less than 2	377	59.5
	2 and above	253	40.5

ANC, antenatal care; PNC, postnatal care.

### Prevalence of cultural malpractices during pregnancy, childbirth, and the postnatal period among women who gave birth once in Dire Dawa in 2021

**Figure 1:**
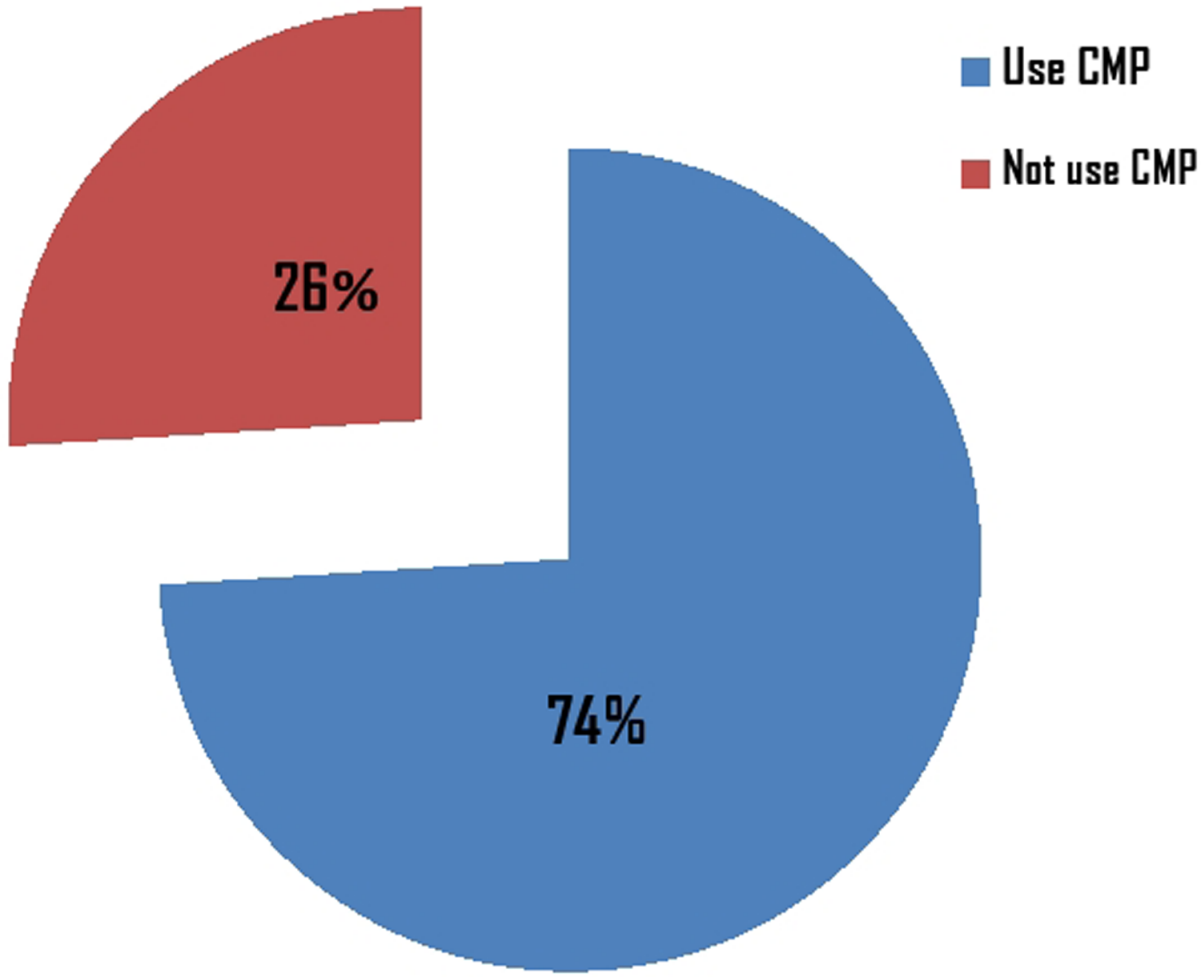
The overall prevalence of cultural malpractices during pregnancy, childbirth, and the postnatal period among women who gave birth once in Dire Dawa city in 2021 (*N* = 624). *CMP, cultural malpractice.

Out of the 624 respondents, 74.4% [95% CI: 70.59%, 77.49%] of the pregnant women were involved in cultural malpractices during their pregnancy, childbirth, and the postnatal period (Figure 1).

### Cultural malpractices during pregnancy

**Figure 2:**
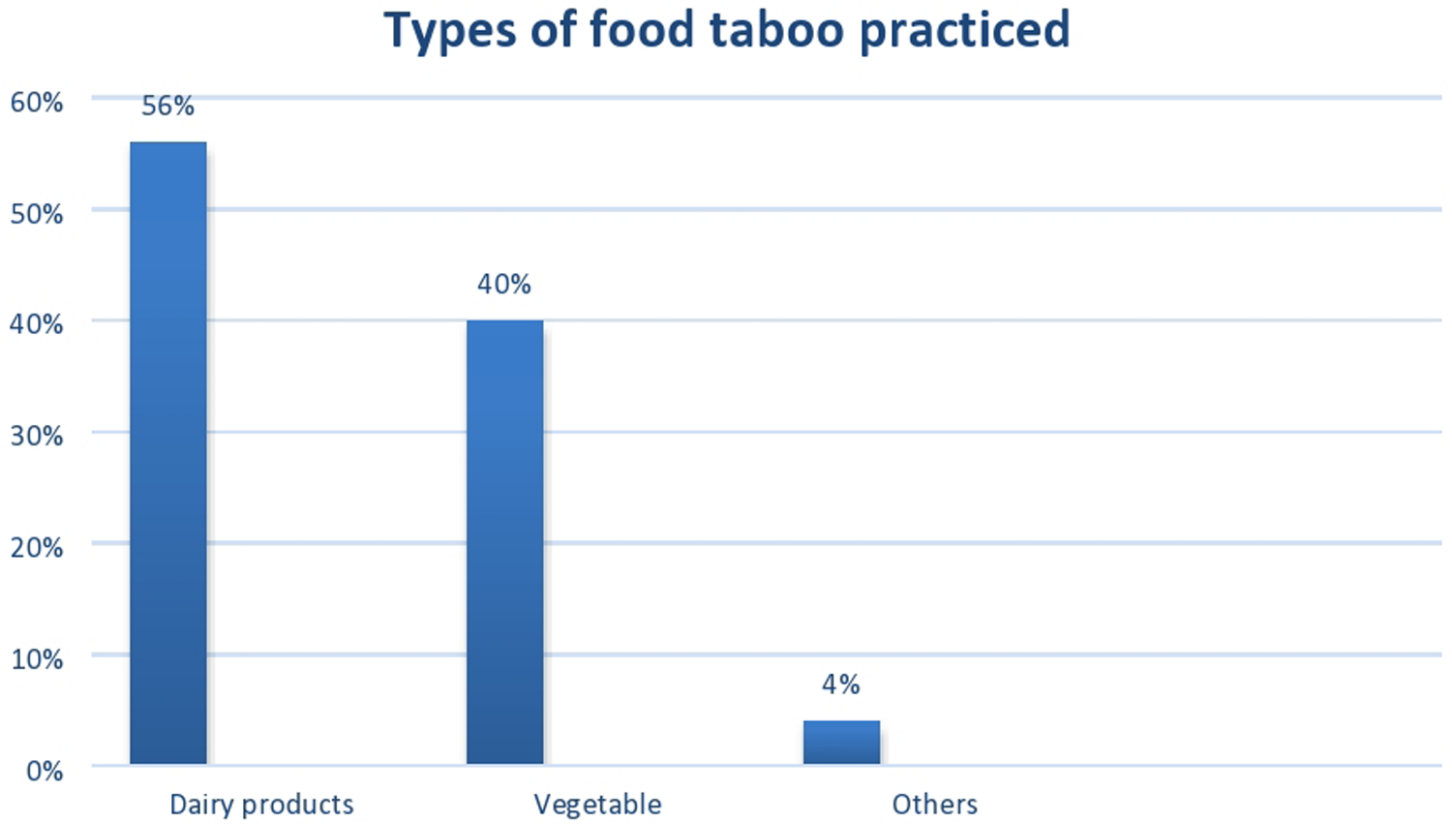
Types of food taboos practiced by women who gave birth once during the pregnancy period in dire dawa city in 2021 (*N* = 624).

In this study, 318 (51.1%) participants reported that they undertook some form of harmful cultural practices during the perinatal period. Regarding food taboos, 50 (8%) of the participants had consumed food taboos (Figure 2). On the other hand, 287 (46%) had drunk *telba* during their pregnancy, and approximately 30 (4.8%) of them had practiced abdominal massage (Table 3).

**Table 3:** Cultural malpractices during the pregnancy period among women who gave birth once in Dire Dawa city in 2021 (*N* = 624).

Characteristics		Frequency	Percent
Nutritional taboos	Yes	50	8
	No	574	92
Types of nutritional taboos (*n* = 50)	Dairy product	28	56
	Vegetable	20	40
	Others	2	4
Abdominal massage	Yes	30	4.8
	No	594	95.2
Drinking *Telba*	Yes	287	46
	No	337	54
Drinking *kosso*	Yes	13	2.1
	No	611	97.9

### Cultural malpractices during childbirth

In terms of birthplace, 96 (15.4%) of the participants had home delivery. Regarding umbilical cord care, 63 (65.6%) respondents used an unclean blade to cut the umbilical cord, and 89 (92.7%) of them used unclean thread to tie the umbilical cord. On the other hand, 44 (45.8%) of the mothers wash their babies before 24 h and 23 (23.9%) of the respondents wash their babies after 24 h (Table 4).

**Table 4:** Cultural malpractices during childbirth among women who gave birth once in Dire Dawa city in 2021 (*N* = 624).

Characteristics		Frequency	Percent
Place of birth	Home	96	15.4
	Health facility	528	84.6
Delivery assisted by whom? (*n* = 96)	TTBA	77	80.2
	UTBA	19	19.8
The method used to expel the placenta	Abdominal massage	14	14.6
	Spontaneously	82	85.4
The initial time of bathing	Before 24 h	73	76
	After 24 h	23	24
The instrument used to cut the umbilical cord	Boiled new blade	33	34.4
	Unboiled new blade	63	65.6
Umbilical cord tied	Yes	96	100
	No	0	0
The material used to tie the umbilical cord	Boiled thread	7	7.3
	Unboiled thread	89	92.7

### Cultural malpractices during the postnatal period

In this study, 259 (41.5%) had been involved in cultural malpractices during their postnatal period. Among these, 131 (21%) of them had applied traditional medicine to the neonate's umbilicus, 89 (67.9%) of the participants applied butter, and 40 (30.5%) of them applied *vaseline or zeyit to* the neonates’ umbilicus during the postnatal period (Table 5).

**Table 5:** Cultural malpractices during the postnatal period among women who gave birth once in Dire Dawa city in 2021 (*N* = 624).

Characteristics		Frequency	Percent
Traditional things applied on the stump of the umbilicus	Yes	131	79
	No	493	21
Types of traditional things applied on the stump of the umbilicus (*n* = 131)	Cow dung	2	1.5
	Butter	89	67.9
	Vasline, zeyit	30	22.9
	Others(herbal medicine	10	7.6
What was given to the child immediately after delivery	Mother's milk	533	85.4
	Other	91	14.6
Time of your babies start breastfeeding	Before 24 h	517	82.9
	After 24 h	107	17.1
The baby was fed colostrum	Yes	551	88.3
	No	73	11.7

### Health system and social-related characteristics of study participants

Regarding the health system and social-related characteristics, 173 (27.7%) of the participants responded that the maternal and child health service was not accessible, and 377 (60.4%) of the participants reported the absence of home-to-home education. Regarding the culture of the participants, 259 (41.5%) of the participants reported that their culture permits those harmful traditional practices (Table 6).

**Table 6:** Health system and social-related characteristics of respondents to assess the prevalence of cultural malpractices during pregnancy, childbirth, and the postnatal period among women who gave birth once in Dire Dawa city in 2021 (*N* = 624).

Characteristics		Frequency	Percent
Accessibility of Maternal and child service	Yes	451	72.3
	No	173	27.7
Home-to-home health education	Yes	247	39.6
	No	377	60.4
Decision on birthplace	Mine	387	62
	Husband	10	1.6
	Me and husband	197	31.6
	Others	30	4.8
Husband supports you during your perinatal period	Yes	456	73.1
	No	168	29.6
Communities permit cultural malpractice	Yes	259	41.5
	No	365	58.5

### Factors associated with cultural malpractice during pregnancy, childbirth, and the postnatal period among women who gave birth once in Dire Dawa city in 2021

In this study, the age of study participants was significantly associated with cultural malpractices during the perinatal period. The odds of cultural malpractices during the perinatal period were two [AOR 2.61, 95%CI, 1.45–4.72] times higher in women ages 35 and above than in women within the age categories of 15–24 and 25–34.

This study also revealed that ANC follow-up was significantly associated with cultural malpractices during the perinatal period. Participants who had not attended ANC follow-up during the last pregnancy were three [AOR 3.57, 95%CI, 1.72–7.40] times more likely to commit cultural malpractices during the perinatal period than their counterparts.

Home-to-home education was another factor that was significantly associated with cultural malpractices during the perinatal period in the study area. Women who did not have home-to-home education were almost two [AOR 1.83, 95%CI, 1.25–2.67] times more likely to commit cultural malpractices than their counterparts.

Lastly, community culture was significantly associated with cultural malpractices during the perinatal period in the study area. Women whose culture (community) permits or allows harmful traditional practices were almost two [AOR 1.69, 95%CI, 1.29–2.54] times more likely to perform cultural malpractices than their counterparts (Table 7).

**Table 7:** Results of bivariate and multivariate analyses for assessment of cultural malpractice during pregnancy, childbirth, and the postnatal period and its associated factors among women who gave birth once in Dire Dawa city in 2021 (*N* = 624).

Variables		Cultural malpractice		COR (95%CI)	AOR (95%CI)	*P*- value
		Yes	No			
Age	15–24	81 (69.8%)	35 (30.2%)	1		
	25–34	204 (66.9%)	101 (33.1%)	0.87 (0.54,1.38)	.96 (.59, 1.55)	0.868
	≥35	77 (87.2%)	26 (12.8%)	2.94 (1.66, 5.20)	**2.62** (**1.45, 4.72)**	**0**.**001**
Educational status	Non-formal education	106 (86.2%)	17 (86.2%)	2.91 (1.65, 5.12)	1.45 (.77, 2.73)	0.243
	Primary (1–8)	142 (75.9%)	45 (24.1%)	1.47 (0.97, 2.22)	0.81 (.50, 1.32)	0.414
	Secondary and above	214 (68.2%)	100 (31.6%)	1		
Occupational status	Housewife	273 (35.6%)	83 (23.3)	1		
	Government employed	73 (62.9%)	43 (37.1)	0.51 (0.32, 0.80)	.64 (0.4, 1.03)	0.068
	Self-employed	116 (76.3%)	36 (23.7%)	0.98 (0.62,1.53)	1.03 (0.64, 1.68)	0.882
ANC follow-up	Yes	366 (70.5%)	153 (29.5)	1		
	No	96 (91.4%)	9 (8.6%)	4.45 (2.19,9.05)	**3.57** (**1.72, 7.40)**	**0**.**001**
Home-to-home health education	yes	116 (67.2%)	81 (32.8%)	1		
	No	296 (78.5%)	81 (21.5%)	1.78 (1.24,2.56)	**1.83** (**1.25, 2.67)**	**0**.**002**
Husband support	Yes	318 (69.7%)	138 (30.3%)	1		
	No	24 (14.3%)	144 (85.7%)	2.6 (1.61,4.19)	1.42 (.84, 2.42)	0.184
Community permit CMP	Yes	213 (82.2%)	46 (17.8%)	2.15 (1.46, 3.17)	**1.69** (**1.12, 2.54)**	**0**.**011**
	No	249(68.2%)	116(31.8%)	1		

ANC, antenatal care; AOR, adjusted odds ratio; CMP, cultural malpractice; COR, crude odds ratio.

The bold value shows that variables that are significantly associated with the Dependent variables.

## Qualitative results

### Study results

A total of 14 participants participated in 11 in-depth interviews and three key informant interviews. The in-depth interview participants comprised 11 women who had histories of giving birth, and the key informant interviews included community and religious leaders. The participants represented a wide age range (19–55 years); most did not attend formal education, and participants were enrolled in farming and were housewives.

From the qualitative analysis of data, four major themes were derived. Participants were interviewed and socio-demographic characteristics, experience of cultural malpractices, types and reasons for cultural malpractices, and availability of health education were discussed. While the themes were reported as being discrete, there was considerable overlap among them. Furthermore, participants' responses to interview questions often addressed more than one theme. In those cases, the interview data were described where they appear to fit most logically.

### Types of cultural malpractices

The period of labor and delivery is embedded with different beliefs, customs, and rituals in different societies that contribute a lot to maternal death. Major cultural malpractices during pregnancy, childbirth, and the postnatal period stated by participants were drinking telba, prohibiting food, using different herbs, and avoiding colostrum. The experience stated by two participants can be read as:

*“During my last period of my pregnancy, my grandmother and my husband advised me to take telba because they told me that it facilitate labor and make my labor easily and short. I have also observed many pregnant mothers in our area while they take telba. I took it starting 7 month up to my labor started”. [sic] (A 32-year-old in-depth interview participant woman)*.

*“In the past three my pregnancies, I have not take meat and egg. Because in our culture meat and egg are not advisable because they may make the baby big and may make my labor difficult”.[sic] (A 37-year-old in-depth interview participant woman)*.

### Cultural influences

Participants describe that cultural beliefs and practices can markedly influence a woman's pregnancy and childbirth experiences and may shape her mothering behavior. In addition, dietary intake before and during pregnancy and through lactation is often influenced by cultural beliefs and practices. The majority of the study participants held the view that the existence of traditional practices and beliefs about food held as taboos are inherent in the community. They stated that the reasons for their practice of cultural malpractices are from cultural influences. Plenty of cultural malpractices directly or indirectly have an impact on the health of the mother and her baby like pre-lacteal feeding, avoidance of colostrum, and restriction of certain food types. One of the participants states her experiences as follows:

*“… Our community strongly believes in culture. Sometimes it may be difficult to be out of the culture of the community. If you fail to do the cultural practice you may have many influences from your friends, families, sometimes you may be discriminated from the society”. [sic] (A 47-year-old Key informant interview participant)*.

### Health education

One of the focus of the study is maternal education and women's empowerment. Participants reveal that there is not much home-to-home health education even at health centers during ANC and PNC. The participants state their experience as follows:

*“….Majority of the time, the health extension comes here for vaccination or registration or for other issue. They does not provide any health education; sometimes they may cone after long period of time. Personally, I believe that the government should provide periodic and regular education on the risks and complications of those harmful cultural practices”. [sic] (A 51-year-old in-depth interview participant woman)*.

“……*In the past 4 of my pregnancy, I have been attended ANC and PNC follow-up. During my ANC follow-up, the ANC room were much crowed and the health professionals are also very busy. They take same sample and do some measurement around my abdomen. Sometimes they may give me some medication. This is a routine experience; they don’t have any time to provide education regarding the CMP*”. [sic] *(A 45-year-old in-depth interview participant woman)*.

## Discussions

The result of the study revealed that the prevalence of cultural malpractices during pregnancy, childbirth, and the postnatal period was 74% [95% CI: 70.59%, 77.49%]. This finding was comparable with studies done in Southern Ethiopia (71.4%) (17), North Karnataka, India (74.5%) (18), Zambia (74.9%) (11), and Cross River State, Nigeria (77.4%) (19). However, the result of this finding was lower than studies done in African women and the Diasporas (79.9%) (8), Turkey (84.5%) (2), Aksum (87.8%) (12), and KwaZulu-Natal, South Africa (79%) (20). It was also higher than studies done in Meshenti (50.9%) (13), Gozamen district (31.2%) (21), and Mizan Aman (35.5%) (7). These findings may differ due to differences in method, study settings, socio-demographic characteristics of study participants, and the availability and accessibility of health service infrastructures.

In this study, women aged 35 and above were more likely to be involved in cultural malpractices during the perinatal period than women aged 15–24 and 25–34. This finding was similar to studies conducted in Mizan aman (7), Gozamen district (21), Southern Ethiopia (17), Nepal (22), Turkey (23), and Nigeria (19). The reason could be that elderly mothers have accepted it as a cultural practice from the past generation.

This study also revealed that participants who had not attended ANC follow-up during their last pregnancy were more likely to be involved in cultural malpractices during the perinatal period than their counterparts. This finding is in agreement with studies conducted in Southern Ethiopia (17), North West Ethiopia (24), Gozamen district (22), Western Region of Ghana (25), Awi zone (26), and Aksum (27). The possible justification for this could be that women who have visited a health facility for an ANC service will have an awareness of the risks and complications of CMP. However, this result was inconsistent with a study done in Turkey (24). This variation might be due to a difference in the availability and accessibility of the health service infrastructure.

Home-to-home health education was another factor that was significantly associated with cultural malpractices during the perinatal period in the study area. Women who did not have home-to-home education were more engaged in cultural malpractices during the perinatal period than their counterparts. *This finding was also supported by the qualitative results.*

“……*In the past 4 of my pregnancy, I have been attended ANC and PNC follow-up. During my ANC follow-up, the ANC room were much crowed and the health professionals are also very busy. They take same sample and do some measurement around my abdomen. Sometimes they may give me some medication. This is a routine experience; they don’t have any time to provide education regarding the CMP*”*.* [sic] *(A 45-year-old in-depth interview participant woman).*

“*…..The majority of the time, the health extension comes here for vaccination or registration or for other issues. They do not provide any health education. Sometimes they may return after a long period of time. Personally, I believe that the government should provide periodic and regular education on the risks and complications of those harmful cultural practices*”*.* [sic] *(a 51-year-old in-depth interview participant woman*).

This finding was consistent with studies conducted in Southern Ethiopia (17), Mizan Aman (7), Raya Kobo (28), the UNICEF report (29), Asia (30), and the United Kingdom (31). The possible reason could be due to the fact that health education is an empowerment tool that not only makes knowledge and information about CMP available but also provides enlightenment as to what courses of action can be taken against CMP. However, this result was inconsistent with a study done in South Africa (21). This variation could be attributed to differences in the study participants' healthcare systems and socio-demographic characteristics.

Lastly, women whose culture (community) permits or allows harmful traditional practices were more engaged in cultural malpractices during the perinatal period than their counterparts.

*This finding was also supported by the qualitative results*.

*“… Our community strongly believes in culture. It can be difficult to be outside of the community's culture at times. If you do not participate in the cultural practice, your friends and family may influence you, and you may face discrimination from society”. [sic] (A 47-year-old key informant interviewed participants).*

This finding was supported by West Gijam (32), Shey Bench (33), Mizan Aman (7), Asia (30), Mbombela City (34), Northern Ghana (35), and Zambia (11). The plausible reason could be that pregnant mothers may be exposed to harmful cultural malpractices because culture has such close attachments to the day-to-day life of a certain society. However, this result was inconsistent with a study done in Australia (2). This demarcation may be attributed to the difference in the socio-economic status of study participants.

## Study implications

As there are few studies conducted on CMPs, this study might have an implication for further research on CMPs and related topics because it can be used as baseline data. It might also have an implication for maternal and child healthcare; by identifying the gaps, it helps to improve quality of care and reduce maternal and child mortality and morbidity.

## Limitations of the study

Despite the fact that well-trained data collectors have been assigned, recall bias may be a concern among mothers with a child less than 12 months before the study to remember what happened to that child's feeding. This was minimized by probing the respondents about the event. Due to the cross-sectional nature of this study, establishing a true cause-and-effect relationship between harmful cultural practices and associated factors would be impossible.

## Conclusions and recommendations

In this study, nearly three-fourths of the study participants were involved in cultural malpractices during the perinatal period. Generally, these findings were almost all higher than other recent studies conducted in Ethiopia so far. Being older than 35, ANC follow-up, home-to-home health education, and the culture of the community were significantly associated with cultural malpractices during the perinatal period.

The Federal Ministry of Health might come up with policies that strengthen community education and behavioral change messages to minimize unhealthy care during pregnancy, childbirth, and the postnatal and neonatal periods, especially with pregnancy at old age (age > 35). Health facilities should provide health education programs that target addressing the major reasons that push mothers to participate in such harmful traditional practices and to increase their maternal and child health (MCH) service utilization, especially ANC. Primary healthcare workers should take an active role in the importance of MCH services during the ANC and PNC follow-ups. Lastly, we would like to recommend that other researchers conduct other study designs for better results.

## Data Availability

The original contributions presented in the study are included in the article/Supplementary Material, further inquiries can be directed to the corresponding author.
